# *Sws*2 Gene Positively Regulates Melanin Production in *Plectropomus leopardus* Skin via Direct Regulation of the Synthesis of Retinoic Acid

**DOI:** 10.3390/ijms25147513

**Published:** 2024-07-09

**Authors:** Haoran Yu, Huapeng Chen, Xinxin Wang, Yichun Zhang, Yafang Tan, Lei Wang, Junlong Sun, Jian Luo, Feibiao Song

**Affiliations:** Collaborative Innovation Center of Nanfan and High-Efficiency Tropical Agriculture, State Key Laboratory of Marine Resource Utilization in South China Sea, Hainan Aquaculture Breeding Engineering Research Center, Hainan Academician Team Innovation Center, School of Marine Biology and Fisheries, Sanya Nanfan Research Institute of Hainan University, Hainan University, Haikou 570228, China; 15666697149@163.com (H.Y.); chenhuapeng2233@163.com (H.C.); 22220951340017@hainanu.edu.cn (X.W.); 13402321117@163.com (Y.Z.); miya772021@outlook.com (Y.T.); wl08092810@163.com (L.W.); 1988sunjunlong@sina.com (J.S.); luojian@hainanu.edu.cn (J.L.)

**Keywords:** *sws*2, skin color, RNAi, retinoic acid, *Plectropomus leopardus*

## Abstract

Opsins are a class of transmembrane proteins encoded by opsin genes, and they play a variety of functional roles. Short wavelength-sensitive opsin 2 (*sws*2), one of the five classes of visual opsin genes, mainly senses blue light. Previous research has indicated that *sws*2 is essential for melanocyte formation in fish; however, its specific role in skin color differentiation remains to be elucidated. Here, we identified the *sws*2 gene in a prized reef-dwelling fish, *Plectropomus leopardus*. The full-length *P. leopardus sws*2 gene encodes a protein consisting of 351 amino acids, and exhibits substantial homology with other fish species. The expression of the *sws*2 gene was widespread across *P. leopardus* tissues, with high expression in eye and skin tissues. Through immunohistochemistry and in situ hybridization analyses, we discovered that the *sws*2 gene was primarily localized in the rod and cone cells of the retina, and epidermal cells of the skin. Furthermore, dsRNA interference was used for *sws*2 gene knockdown in living *P. leopardus* to elucidate its function in skin color differentiation. Black-color-related genes, melanin contents, and tyrosinase activity in the skin significantly decreased after *sws*2 knockdown (*p* < 0.05), but red-color-related genes and carotenoid and lutein contents significantly increased (*p* < 0.05). Retinoic acid injection produced the opposite results. Our results suggested that the *sws*2 gene influences *P. leopardus* skin color regulation by affecting vitamin synthesis and melanin-related gene expression levels. This study establishes a foundation for elucidating the molecular mechanisms by which *sws*2 regulates melanocyte formation in fish skin.

## 1. Introduction

Coloration stands out as one of the most significant traits in fish, and is closely associated with their survival and economic value. Fish exhibit a diversity of color variations, with the regulation of their coloration closely linked to factors such as genetics, physiological status, and their environment [1]. Environmental factors significantly influence fish coloration, with environmental color being one of the most influential factors. Virtually all fish species exhibit camouflage coloration, allowing them to blend in with the environment for protection. Changes in fish coloration in response to changes in environmental color primarily occur through visual mechanisms [2,3,4,5]. In addition, UVR is a major environmental regulator of melanogenesis. Fish can adjust their spectral absorbance by modifying their visual opsin spectrum, thereby better adapting to their environment and increasing their likelihood of survival [5]. For instance, the skin color on the back of fish is mainly dark, because melanogenesis is under complex regulatory control through the interaction of multiple agents, and UVR is a major environmental regulator of melanogenesis [6,7]. The photoreceptors in fish are located in the eyes. The outermost layer of the eye is covered by a thick and tough sclera. The anterior part of the sclera forms the cornea, which contains pigments. Inside the sclera are the silvery layer, choroid, and retina. The photoreceptor cells in fish are the cone cells and rod cells of the retina. Additionally, the skin of fish serves as the display area for body coloration. From the outside inwards, it consists of the epidermis and dermis, which contain numerous pigment cells [8]. Opsins are a class of transmembrane proteins encoded by opsin genes, each containing approximately 350 amino acid residues [9]. Belonging to the GPCR (G protein-coupled receptor) family, they play pivotal roles in diverse aspects of the visual system, including visual image formation, circadian rhythms, regulation of reproductive cycles, and pigment dispersion [10,11,12]. Opsins are categorized as visual or non-visual based on their direct involvement in visual imaging [11,13]. Due to gene duplication events, fish visual systems retain five opsin genes: one in rod cells, *rh*1 (rhodopsin), and four in cone cells, namely *m*/*lws* (middle/long wavelength-sensitive opsin), *sws*2 (short wavelength-sensitive opsin 2), *sws*1 (short wavelength-sensitive opsin 1), and *rh*2 (rhodopsin-like opsin) [14,15]. The various visual proteins in fish are expressed in different sites. For instance, *rh*1 is expressed in rod cells, while *m*/*lws* and *rh*2 are expressed in different regions of double cone cells, and *sws*2 is expressed in long-wavelength single cone cells, while *sws*1 is expressed in short-wavelength single cone cells [11].

Several studies have demonstrated that the differential expression of opsin genes is pivotal in the response to different wavelength stimuli [16,17,18]. The main function of the *sws*2 gene is to perceive light in the blue wavelength range. Given its deep penetration in water, blue light is one of the primary light sources for aquatic life, making it a central factor in the ecological adaptation of aquatic organisms [19]. In this context, research on the *sws*2 gene not only aids in understanding how fish perceive the different spectra in their environment, but also reveals the regulatory mechanisms underlying their ecological, biological, and behavioral adaptations [20]. Previous studies have indicated that variations in the expression and function of the *sws*2 gene significantly impact fish visual behavior and ecological adaptability. For instance, alterations in *sws*2 gene expression have been implicated in regulating fish coloration, visual behavior, and adaptability to environments [21,22,23]. Additionally, studies in half-smooth tongue sole (*Cynoglossus semilaevis*) and olive flounder (*Paralichthys olivaceus*) have shown that the blue opsin gene *sws*2 affects the asymmetric development of coloration in flatfish by promoting the synthesis of retinoic acid [24]. Therefore, in-depth research on the function and regulatory mechanisms of the *sws*2 gene is vital to elucidating the workings of the fish visual system and ecological adaptation mechanisms.

Retinoic acid (RA) refers to a class of chemical substances derived from the conversion of vitamin A within the cells of an organism. It is an important bioactive form of vitamin A in the body and can act as a bioactive regulator, participating in the regulation of various activities such as growth and development, proliferation, differentiation, and apoptosis of multiple tissue structures in different ways [25,26]. In vertebrates, higher levels of retinoic acid correlate with higher deposition of melanin. Injecting a certain concentration of retinoic acid into the mantle edge channel of oysters can cause their shell color to turn black, and there is a positive correlation between the retinoic acid content in the oyster mantle and the melanin content [27]. *Plectropomus leopardus*, a valued species among reef enthusiasts, is commonly found in tropical and subtropical marine habitats. In its natural habitat, *P. leopardus* exhibits a vibrant red or orange-red coloration, and it is characterized as having high economic and aesthetic value. However, in recent years, artificially cultivated *P. leopardus* have undergone changes in coloration due to physiological and/or environmental factors, gradually darkening in hue, which has significantly impacted their economic worth. Our previous research indicated that white and blue backgrounds maintain the red coloration of juvenile *P. leopardus*, while black, red, and transparent backgrounds cause them to darken [28]. However, our findings from another study indicated that *P. leopardus* reared in environments with different background colors exhibited similar iris and skin colors [29]. A qPCR analysis revealed that there was significant differential expression of the *sws*2 gene among different groups (*p* < 0.05), suggesting that the *sws*2 gene is involved in mediating color changes in *P. leopardus* through visual mechanisms.

Building upon these observations, the objective of the present study was to explore the function and molecular mechanisms of the *sws*2 gene in mediating color changes in *P. leopardus*. Therefore, we first cloned the full-length sequence of the *sws*2 gene and employed fluorescence qRT-PCR, immunohistochemistry, and fluorescence in situ hybridization techniques to analyze its expression in tissue and its subcellular localization. Additionally, we utilized in vivo RNA interference techniques to assess the functional role of the *sws*2 gene in color changes in *P. leopardus*. Finally, through the intravenous injection of retinoic acid via the caudal vein, we conducted a systematic examination of alterations in the expression of *sws*2 and genes related to coloration, as well as variations in pigment-related enzyme activity, to elucidate the molecular mechanisms by which the *sws*2 gene regulates color changes.

## 2. Results

### 2.1. P. leopardus sws2 cDNA Cloning and Sequence Analysis

**Figure 1:**
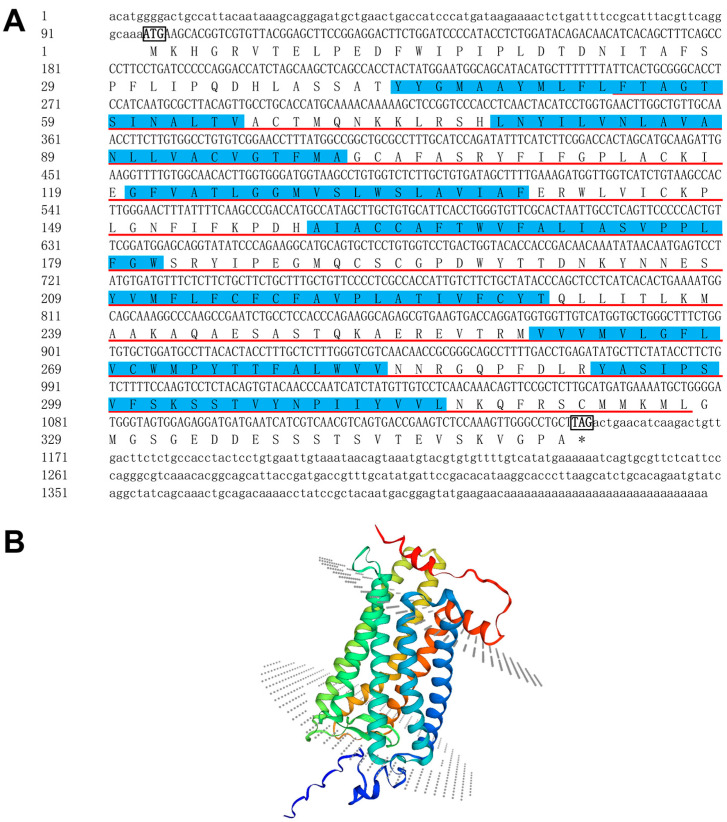
Bioinformatics analysis of *P. leopardus sws*2. (**A**) The start codons (ATG) and stop codons (TGA) are boxed and bolded; the red underlined sequences are the 7TM GPCR srsx domain and the blue shadow identifies the transmembrane sequence. (**B**) Homology-based 3D model of Sws2.

The *P. leopardus sws*2 cDNA comprised 1438 bp nucleotides which encodes a protein consisting of 351 amino acids, contained an open reading frame (ORF) of 1056 bp, encompassing a 95 bp 5′-untranslated region and a 287 bp 3′-untranslated region, and it also harbors the poly A signal sequence (Figure 1A). The predicted protein had a calculated molecular mass of 38.972 kDa and an isoelectric point of 7.78. This Sws2 protein was made up of serpentine-type 7TM GPCR chemoreceptor srsx (Figure 1A). The homology-based 3D model of Sws2 exhibited the characteristic seven transmembrane structures, as illustrated in Figure 1B.

### 2.2. Multiple Sequence Alignment and Phylogenetic Analysis

**Figure 2:**
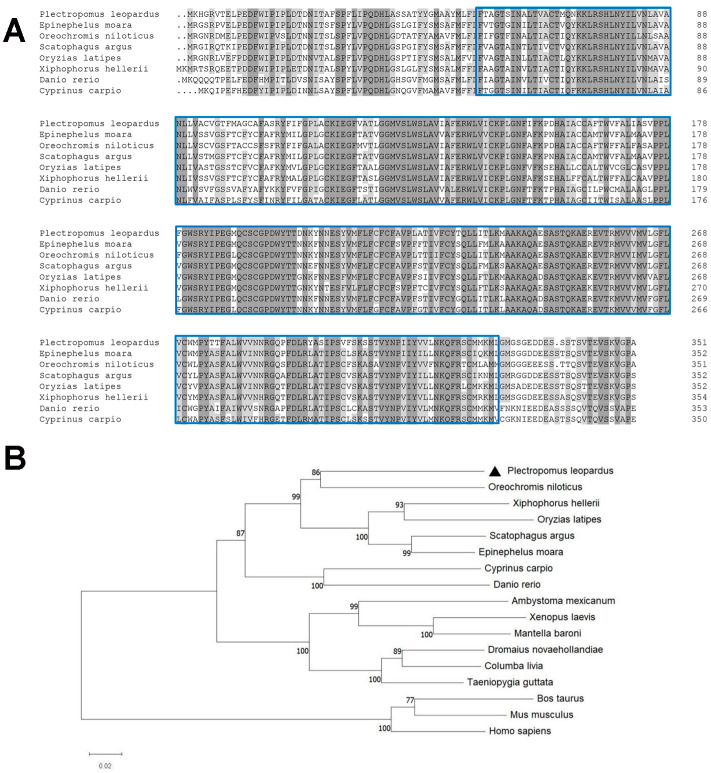
Comparison of Sws2 aa sequences from different species (**A**) and phylogenetic analysis (**B**). (**A**) Dark shaded regions represent instances where species share identical amino acid sites, while light shaded regions indicate sites where over half of the listed species exhibit identical amino acid residues. The blue boxes indicate the 7TM GPCR srsx domain.

The homology of Sws2 between *P. leopardus* and deduced amino acid sequences from other species was conducted using DNAMAN through multiple sequence alignment (Figure 2A). The deduced amino acid sequence of *P. leopardus* Sws2 exhibited a high degree of identity with that of *Epinephelus moara*. Unrooted phylogenetic trees were generated using the neighbor-joining (NJ) method with 1000 bootstrap tests, utilizing multiple amino acid sequence alignments of Sws2 from various vertebrate species. The results showed that the Sws2 of *P. leopardus* first clustered with *Oreochromis niloticus*, then with other species, and finally with mammals (Figure 2B).

### 2.3. Expression Levels of sws2 in Different Tissues

**Figure 3:**
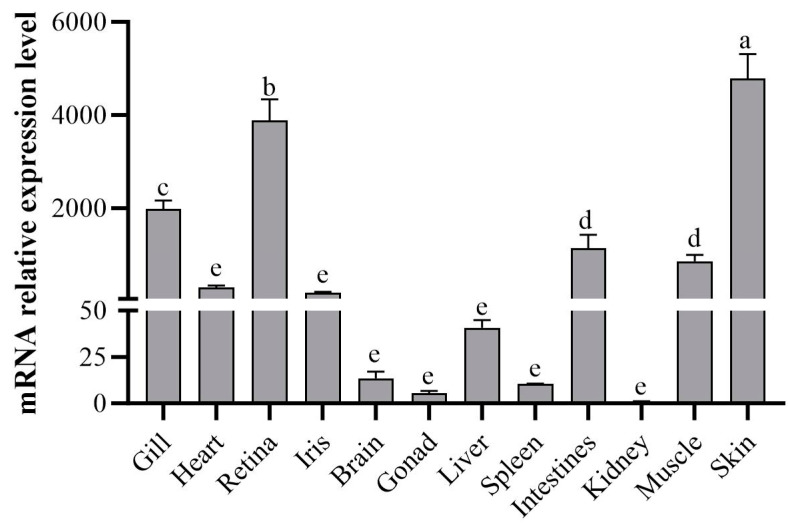
Tissues profile of *sws*2 mRNA expression in *P. leopardus*. Significant differences among the means of experimental groups are denoted by distinct lowercase letters (*p* < 0.05).

The expression levels of *sws*2 in the different tissues of *P. leopardus* were determined using qPCR. As shown in Figure 3, *sws*2 mRNA was widely distributed and found in all tissues of *P. leopardus*. *Sws*2 mRNA was most abundant in the skin (*p* < 0.05), followed by the retina and gill, and the lowest expression was in the kidney and gonad (*p* < 0.05, Figure 3).

### 2.4. Localization of Sws2 in the Eye and Skin of P. leopardus

**Figure 4:**
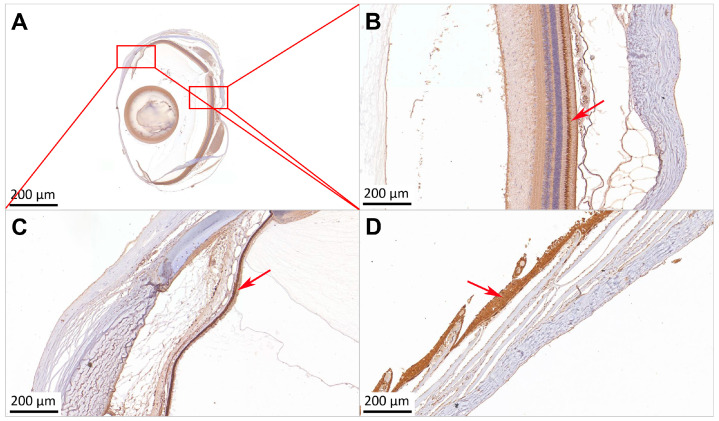
Immunohistochemical localization of Sws2 in *P. leopardus* eye and skin tissues. Brown coloration means positive signals. (**A**): The overall paraffin section of the *P. leopardus* eye. Sws2 was localized in the iris (**C**) and retina (**B**) of the eye, and epidermal cells of the skin (**D**). The red arrow means positive signal.

An immunohistochemical analysis revealed positive Sws2 staining (brown coloration) in both eye and skin tissues. (Figure 4). In the tissues of different parts of the eye, Sws2 was mainly concentrated in the rod and cone cells of the retina (Figure 4B), and iris (Figure 4C). In the skin tissues, Sws2 was mainly concentrated in the epidermal cells (Figure 4D).

### 2.5. In Situ Hybridization of sws2 in the Eye and Skin of P. leopardus

**Figure 5:**
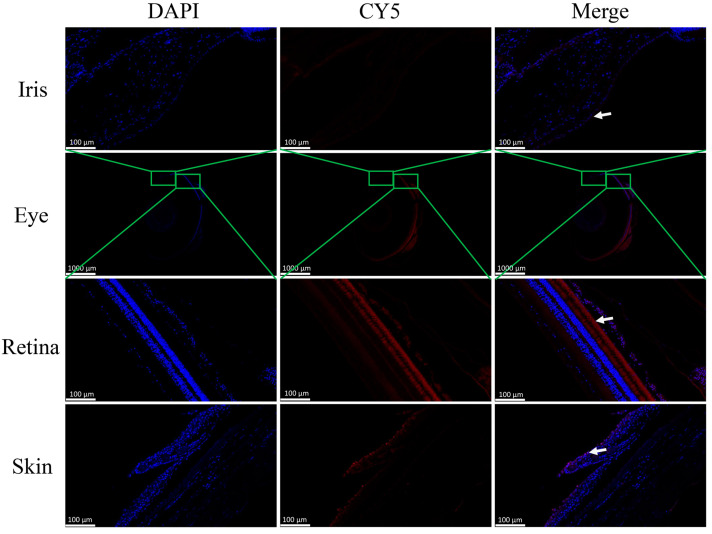
In situ hybridization of the *sws*2 gene in the eye and skin of *P. leopardus*. The white arrow means positive signal.

To further validate the expression locations of the *sws*2 gene, the cellular distributions of *sws*2 mRNA in the eye and skin tissues were characterized via FISH analysis, with the positive signal appearing in red. As shown in Figure 5, *sws*2 was expressed in the iris and retina tissues of the eye, but mainly in the rod and cone cells and mainly in the epidermal cells of the skin, consistent with the immunohistochemistry results.

### 2.6. Alterations in sws2 Expression Levels and Genes Related to Skin Color following sws2 Knockdown

**Figure 6:**
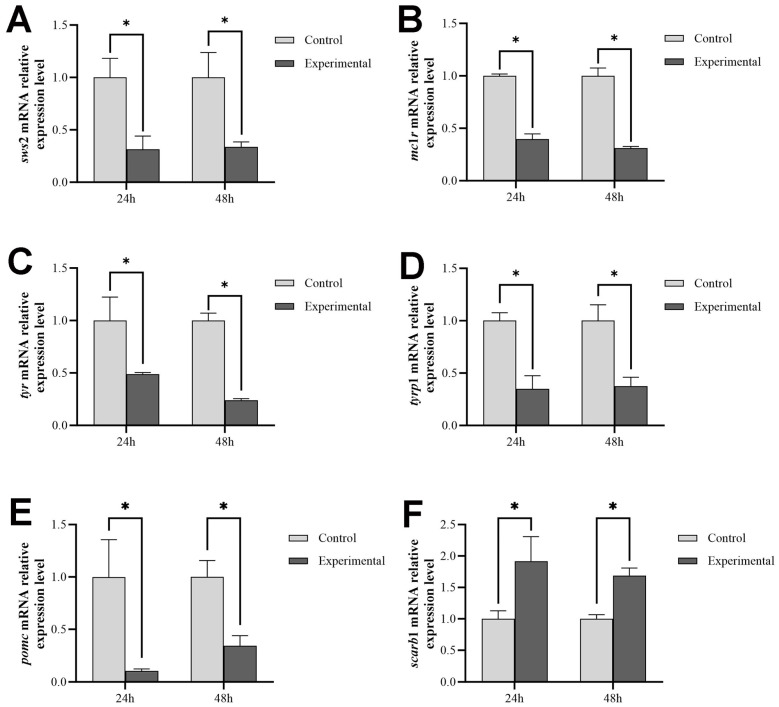
Expression levels of *sws*2 (**A**), *mc*1*r* (**B**), *tyr* (**C**), *tyrp*1 (**D**), *pomc* (**E**), and *scarb*1 (**F**) in the skin of *P. leopardus* after *sws*2 knockdown. The * means significant differences.

As shown in Figure 6, the *sws*2 expression levels significantly decreased after 24 and 48 h of *sws*2 interference (*p* < 0.05), this coincided with notable alterations in the expression levels of genes involved in melanin and carotenoid biosynthesis in the skin of *P. leopardus*. The expression levels of *mc*1*r*, *tyr*, *tyrp*1, and *pomc* significantly decreased (*p* < 0.05) and *scarb*1 significantly increased (*p* < 0.05) under *sws*2 interference.

### 2.7. Pigment Content and Tyrosinase Activity after sws2 Knockdown

**Figure 7:**
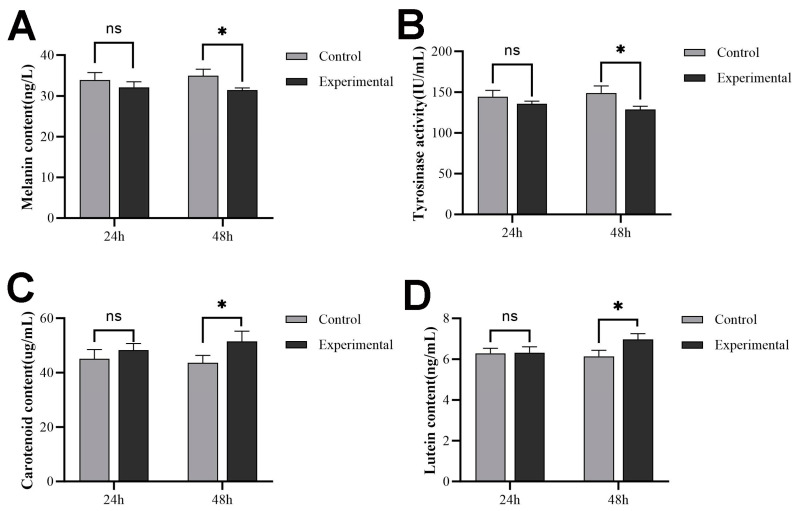
Pigment content and tyrosinase activity in the skin of *P. leopardus* after *sws*2 knockdown. (**A**) Melanin content of *P. leopardus* after *sws*2 knockdown; (**B**) tyrosinase activity of *P. leopardus* after *sws*2 knockdown; (**C**) carotenoid content of *P. leopardus* after *sws*2 knockdown; (**D**) lutein content of *P. leopardus* after *sws*2 knockdown. The * means significant differences, ns means no significant differences.

The pigment content and tyrosinase activity in fish skin changed significantly at 48 h after *sws*2 knockdown. As depicted in Figure 7, the melanin content in the skin showed no significant alteration at 24 h, but decreased significantly at 48 h (Figure 7A, *p* < 0.05). The trends in tyrosinase activity mirrored those of the melanin content (Figure 7B). In addition, the carotenoid and lutein contents in the skin both had no significant change at 24 h, then significantly increased after *sws*2 knockdown (*p* < 0.05) (Figure 7C,D), with a greater increase in the carotenoid content.

### 2.8. Expression Levels of Skin-Color-Related Genes after Retinoic Acid Treatment

**Figure 8:**
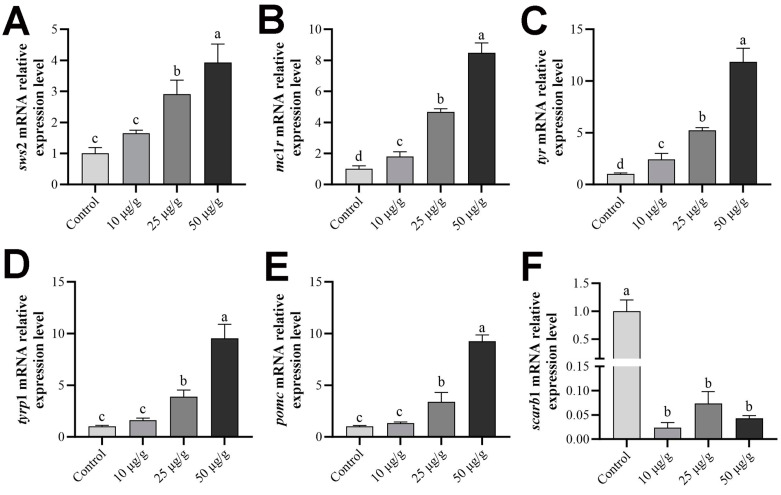
Expression levels of *sws*2 (**A**), *mc*1*r* (**B**), *tyr* (**C**), *tyrp*1 (**D**), *pomc* (**E**), and *scarb*1 (**F**) in the skin of *P. leopardus* after retinoic acid treatment. Significant differences among the means of experimental groups are denoted by distinct lowercase letters (*p* < 0.05).

As shown in Figure 8, there were significant differences in the expression levels of melanin and carotenoid biosynthesis-related genes in the skin of *P. leopardus* following retinoic acid treatment. The expression levels of *sws*2 (Figure 8A), *mc*1*r* (Figure 8B), *tyr* (Figure 8C), *tyrp*1 (Figure 8D), and *pomc* (Figure 8E) significantly increased after retinoic acid intravenous injection, with the highest increases occurring in the 50 μg/g group (*p* < 0.05), and *scarb*1 significantly decreased (*p* < 0.05; Figure 8F).

### 2.9. Pigment Content and Tyrosinase Activity after Retinoic Acid Treatment

**Figure 9:**
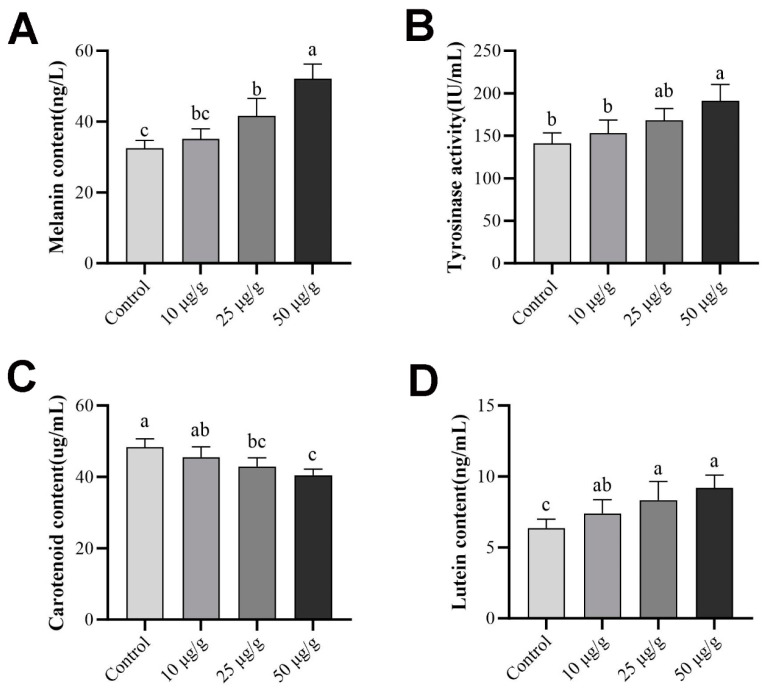
Pigment content and tyrosinase activity in the skin of *P. leopardus* after retinoic acid treatment. (**A**) Melanin content after retinoic acid treatment; (**B**) tyrosinase activity after retinoic acid treatment; (**C**) carotenoid content after retinoic acid treatment; (**D**) lutein content after retinoic acid treatment. Significant differences among the means of experimental groups are denoted by distinct lowercase letters (*p* < 0.05).

The pigment melanin and lutein contents and tyrosinase activity in *P. leopardus* skin changed significantly after retinoic acid intravenous injection (*p* < 0.05). As shown in Figure 9, the melanin content and tyrosinase activity increased with higher concentrations of retinoic acid, with a significant increase observed in the 50 μg/g group (Figure 9A,B, *p* < 0.05). Conversely, the carotenoid content exhibited a decreasing trend with increasing concentrations of retinoic acid, with a significant decrease observed in the 50 μg/g group (Figure 9C, *p* < 0.05). What is puzzling is that, the lutein content also showed a downward trend, similar to that of melanin.

## 3. Discussion

Visual pigment proteins have evolved considerable diversity under natural selection, with many fish genomes containing one to three copies of *sws*2 gene [27,28,29,30,31,32,33]. For example, *Oryzias latipe*s contains two orthologous genes of *sws*2, namely *sws*2a and *sws*2b [34]. In the present study on *P. leopardus*, only one full-length cDNA of the *sws*2 gene was cloned and analyzed, possibly due to loss during the genome replication process. The *P. leopardus* Sws2 amino acid sequence shared the highest similarity (93.16%) with that of *Epinephelus lanceolatus* and *Epinephelus fuscoguttatus*. The Sws2 protein contains a domain that is unique to the opsin gene family, the serpentine-type 7TM GPCR chemoreceptor srsx, a class of integral membrane proteins that contain seven membrane-spanning helices. The phylogenetic analysis indicated that Sws2 protein first clustered with *Oreochromis niloticus*, then with other fish species, showing that *sws*2 has indeed been highly conserved during evolution [35].

We initially examined the expression pattern of *sws*2 in *P. leopardus* tissues. The *sws*2 gene was detected in all tissues and exhibited high expression levels in eye and skin tissues, indicating its significant involvement in skin color changes. Similarly, among tissues in adult *Oryzias latipes* and *Lucania goodei*, *sws*2 was expressed mainly in the eye [34,36]. Measurements of opsin sequences and opsin expression are frequently employed to deduce fundamental characteristics of visual signaling and the visual system [16,37,38]. The expression of *sws*2 in eye tissue likely contributes significantly to the absorption of blue light and color vision. However, previous research has paid relatively little attention to the expression of opsin genes in the skin of fish. In this study, we also found that the *sws*2 gene was highly expressed in the skin, potentially indicating that *P. leopardus* skin can sense and respond to blue light. Our previous studies showed that light color and background color had significant effects on *P. leopardus* skin color; therefore, we inferred that the *sws*2 gene may play an important role in skin color changes in *P. leopardus*.

Visual pigments, which comprise opsin receptor proteins in photoreceptor cells, are crucial for light detection and signal transduction. Opsins play a key role in mediating light absorption in both cones and rods [39,40,41]. Immunohistochemistry and in situ hybridization revealed that the *P. leopardus sws*2 gene was mainly concentrated in iris and retina tissues of the eye, especially in the rod and cone cells of the retina. Previous studies indicated that in vertebrates, *sws*2 is a G-protein-coupled receptor expressed in cone cells within the retina, and it is responsible for vision in bright or well-lit conditions [36,42,43]. This is consistent with our study, which indicated that *P. leopardus* is more sensitive to blue light. Interestingly, we also found that the *sws*2 gene is expressed in the epidermal cells of the skin. The epidermis of *P. leopardus* contains a large number of pigment cells, which play an important role in regulating skin color changes. Thus, we speculated that *sws*2 gene expression in *P. leopardus* skin can affect skin color changes by sensing environmental colors.

To verify the effect of *sws*2 gene expression on *P. leopardus* skin color changes, dsRNA interference technology was used to knockdown the *sws*2 gene in living *P. leopardus*. The expression of black-color-related genes, melanin contents, and tyrosinase activity in the skin all significantly decreased after *sws*2 knockdown (*p* < 0.05), while the expression of red-color-related genes and carotenoid and lutein contents both significantly increased (*p* < 0.05). Carotenoids are also one of the main pigments that affect the color characteristics of fish. In *Dawkinsia filamentosa*, the black background tank helped body pigmentation and promoted carotenoid accumulation [44]. And, the carotenoid content was regulated by *scarb*1 (scavenger receptor class B type I) [45,46]. The *scarb*1 gene is involved in the selective absorption and transport of carotenoids, and this function is conservative among different species [47]. Studies in zebrafish have demonstrated that UV light can impact the expression of *raldh*2 and the synthesis of retinoic acid through *sws*1, subsequently influencing the expression of *pomc* to regulate melanocyte formation [48]. Cone opsins of the *sws*1 class typically generate photopigments with λ_max_ values ranging from 360 nm to 450 nm, while *sws*2 photopigments exhibit λ_max_ values between 400 nm and 470 nm. These spectral ranges are based on photopigments containing a vitamin-A1-derived chromophore [49], potentially upregulating the synthesis of vitamin A1. Vitamin A1 is mainly metabolized by alcohol dehydrogenase to retinal and then to retinoic acid. Therefore, we speculated that the *sws*2 gene influences skin color regulation in *P. leopardus* by affecting vitamin synthesis and the expression of melanin-related genes, positively regulating black color genes, and negatively regulating red color genes.

In order to further validate our hypothesis, retinoic acid was injected into *P. leopardus* via the tail vein. The results showed that the expression of black-color-related genes, melanin contents, and tyrosinase activity in the skin increased with the increase in retinoic acid concentration, while the *scarb*1 and carotenoid content significantly decreased (*p* < 0.05); these are consistent with observations in *Paralichthys olivaceus* and *Crassostrea gigas* [24,27]. These results indicated that the blue-sensitive opsin gene *sws*2 in the skin of *P. leopardus* induces the synthesis of retinoic acid in the skin after receiving blue light, thereby promoting the formation of melanocytes and the subsequent blackening of *P. leopardus* skin color. This study further indicated that the color changes in fish skin are regulated by multiple pathways, and also provided a basis for understanding the molecular mechanism by which *sws*2 regulates melanocyte formation in fish skin.

Despite the significant findings of this study, several limitations warrant attention. First, while the *sws*2 gene’s role in skin color differentiation in *P. leopardus* was established, the precise molecular pathways remain inadequately understood. The mechanisms through which *sws*2 influences vitamin synthesis and melanin-related gene expression are complex and need further exploration. Additionally, the study primarily focused on the gene’s effect on melanin and carotenoid pathways, but other potential pathways and factors influencing skin color were not comprehensively examined. Future research should aim to delineate the downstream targets and interacting partners of *sws*2 to provide a more holistic understanding of its function.

## 4. Materials and Methods

### 4.1. Fish

The experimental juvenile *P. leopardus* weighed approximately 10 g at four months post-hatching, and were provided by Hainan Leyu Technology Co., Ltd. (Chengmai, China). During both the acclimation and experimental periods, the fish were reared in indoor tanks measuring 0.8 m × 0.8 m × 1 m, with a water height of 0.8 m. They were subjected to a natural light/dark photoperiod of 12 h at a constant temperature of 28 ± 1 °C. Feeding occurred three times daily (08:00, 12:00, and 17:00) using a compound feed (Guangdong Yuequn Biotechnology Co., Ltd., Jieyang, China).

### 4.2. Fish Sampling, RNA Extraction, and First-Strand cDNA Synthesis

The experimental fish underwent anesthesia using MS-222, followed by the collection of skin and eye tissues. Half of each sample was rapidly frozen in liquid nitrogen and stored at −80 °C until further processing, while the remaining half was fixed in 4% paraformaldehyde. The total RNA was extracted from the samples using the Trizol reagent (Promega, Madison, WI, USA) following the manufacturer’s protocol. Genomic DNA was eliminated using RNase-free DNase (Takara, Otsu, Japan). The concentration, quality, and integrity of the total RNA were assessed using a UV spectrophotometer (NanoDrop 2000, Thermo, Wilmington, DE, USA) by measuring the OD 260/280 ratio, and through 1% agarose gel electrophoresis.

The first-strand cDNA was synthesized using the PrimeScript RT Master Mix (Takara) for standard cDNA synthesis. Additionally, for the synthesis of 5′ and 3′ end cDNA (5′ and 3′ RACE), a SMARTer RACE 5′/3′ Kit (Clontech, Mountain View, CA, USA) was employed with skin RNA. Following synthesis, the reaction product was diluted with Tricine–EDTA buffer to a final volume of 90 μL. All resulting cDNA samples were then stored at −20 °C and utilized as PCR templates as needed.

### 4.3. Molecular Cloning and Bioinformatics Analysis

The 5′/3′-RACE reaction was conducted using the RACE cDNA Amplification Kit (Takara) following the manufacturer’s instructions. Specific primers for sws2 gene RACE PCR are detailed in Table 1. The PCR conditions were as follows: 94 °C 3 min, 40 cycles for 30 s at 94 °C, then 59 °C for 30 s, and extend for 2 min at 72 °C. The nested PCR conditions were as follows: 35 cycles at 94 °C for 30 s, 58 °C for 30 s, and 72 °C for 2 min. Purification of the PCR products was carried out using the E.Z.N.A^®^ Gel Extraction Kit (Omiga BioTek, Norcross, GA, USA). Subsequently, the purified PCR products were ligated into the pMD^®^-19 T vector (TaKaRa) overnight and transformed into Escherichia coli DH5-alpha competent cells for sequencing.

The obtained nucleotide and deduced amino acid (AA) sequences were subjected to analysis using BLAST (https://blast.ncbi.nlm.nih.gov/Blast.cgi accessed on 21 October 2023) and Clustal Wtools (https://www.ebi.ac.uk/jdispatcher/ (accessed on 21 October 2023)). The open reading frame (ORF) of *sws*2 was predicted using Open Reading Frame Finder. Tertiary structure analysis was conducted using the online tool SWISS-MODEL (https://swissmodel.expasy.org/interactivel.expasy.org/interactive accessed on 21 October 2023). Additionally, a neighbor-joining (NJ) phylogenetic tree was constructed utilizing MEGA 5.0.

### 4.4. qRT-PCR (Quantitative Real-Time PCR) Analysis

To determine the mRNA expression levels, the qRT-PCR method was utilized, with primers designed based on the nucleotide sequences from *P. leopardus* (Table 1). PrimeScript RT Master Mix (Takara) was employed for the synthesis of *sws*2 first-strand cDNA. Subsequently, a CFX96 qPCR System (Bio-Rad, Hercules, CA, USA) was used for qRT-PCR analysis using SYBR Premix Ex TaqII (Takara). The thermal cycling conditions included an initial denaturation step at 95 °C for 30 s, followed by 40 cycles of denaturation at 95 °C for 5 s and annealing/extension at 58 °C for 30 s. The comparative CT (2^−ΔΔCt^) method was used to calculate gene expression levels, with each sample analyzed in triplicate alongside the internal reference gene.

### 4.5. Immunohistochemistry

For the eye and skin samples, 5 μm thick sections were prepared in paraffin and placed into a constant-temperature box and dried at 68 °C for 90 min. The paraffin sections underwent deparaffinization and hydration, and were sequentially immersed in xylene I, xylene II, xylene III, absolute ethanol, 90% ethanol, 80% ethanol, and 70% ethanol. Then, the sections were rinse three times with distilled water for 3 min, followed by washing with PBS three times for 3 min. Subsequently, slides with the sections were slowly submerged in boiling EDTA (pH 9.0), heated in a microwave oven set at 100 °C for 10 min, and allowed to cool naturally for approximately 20 min to return to room temperature and removed from the oven. The slides were then rinsed three times with PBS buffer solution for 5 min. Following this, 3% hydrogen peroxide and goat serum blocking solution were added separately and the slides were incubated at room temperature for 10 min to deactivate endogenous enzyme activity. The blocking solution was then removed, and Sws2 rabbit monoclonal antibody (1:100) was added and left to incubate overnight at 4 °C. Subsequently, the sections were washed three times with PBS and distilled water. Afterwards, the secondary antibody was added and incubated at room temperature for 10 min. After washing, the sections were incubated with DAB chromogenic solution. The samples were observed under a fluorescence microscope, and staining was terminated when appropriate, followed by rinsing with tap water. Hematoxylin counterstaining and dehydration were performed, followed by photography under a fluorescence microscope. Normal serum was used instead of the primary antibody as a negative control.

### 4.6. Fluorescence In Situ Hybridization

The probes and primers were designed based on the *sws*2 gene sequence and synthesized by Guangzhou Bersinbio Biotechnology Co., Ltd. (Guangzhou, China). (Table 1). The prepared paraffin sections were deparaffinized by immersion in xylene three times for 6 min each. Subsequently, they were dehydrated in a series of gradient ethanol solutions (70%, 80%, 90%, and 100%) for 5 min each and air-dried at room temperature. The samples were treated with 20 microliters of prehybridization solution, covered, and incubated at 37 °C for 30 min to prevent non-specific binding. A hybridization reaction mixture containing probes and hybridization solution at a ratio of 1:39 was prepared, thoroughly mixed, and quickly placed on ice. Thirty microliters of the hybridization reaction mixture was added to the samples, which were covered and incubated at 73 °C for 6 min for denaturation, followed by rapid transfer to 42 °C for overnight hybridization. After washing, 20 μL of DAPI was added to the samples, which were covered and stained in the dark for 10 min. The samples were washed twice with 1 × PBS for 5 min and air-dried in the dark. Finally, 15 μL of anti-fade mounting medium was added to seal the slides, and fluorescent images were captured using a fluorescence microscope. The target gene *sws2* exhibited red fluorescence (CY5).

### 4.7. Effect of Sws2 Knockdown on Skin Color

The sws2 dsRNA amplification primers were designed utilizing the online tool SnapDragon-dsRNA Design for primer design (https://www.flyrnai.org/snapdragon (accessed on 21 October 2023)) (Table 1), and *sws*2 dsRNA was synthesized using the TranscriptAid T7 High Yield Transcription Kit (Thermo Scientific) according to the manufacturer’s instructions. Juvenile *P. leopardus*, averaging 9.02 ± 0.20 g in body weight, were randomly allocated into either the experimental or control group, with three replicates in each group and 20 fish per replicate. The experimental group received intraperitoneal injections of 5 μg *sws*2 dsRNA per g body weight, while the control group was injected with an equal volume of H_2_O. On the 24th and 48th hour after injection, the fish were anesthetized with MS-222, after which, skin and eye tissues were collected, immediately snap-frozen in liquid nitrogen, and stored at −80 °C until further processing. The expression levels of skin-color-related genes were detected by qPCR, and the levels of pigments, MSH content, and tyrosinase activity were assessed using ELISA kits (Zhenke Industrial International, Zhongshan, China) in accordance with the manufacturer’s instructions.

The skin was washed with precooled PBS (0.01 M, pH 7.4), weighed, homogenized with cold PBS, and centrifuged (2000× *g* for 20 min) to obtain the supernatant. Standard and sample wells, as well as blank wells, were set up on an antibody-coated plate. To each well, 50 μL of varying concentrations of standard solution, 40 μL of Sample Diluent, and 10 μL of sample were added. A total of 100 μL of HRP Conjugate was added to all wells except the blank wells. The plate was incubated at 37 °C for 60 min, washed five times, and then 50 μL of Chromogen Solution A and 50 μL of Chromogen Solution B were added. After incubating at 37 °C for 15 min in the dark, 50 μL of Stop Solution was added to each well. The color change from blue to yellow was measured at 450 nm within 15 min. The blank well served as the zero reference.

### 4.8. Retinoic Acid Treatment

Juvenile *P. leopardus*, averaging 20.01 ± 0.53 g in body weight, were randomly allocated into one control group and three treatment groups. Each group comprised three tanks, with 15 fish housed in each tank. The treatment groups received a single dose of retinoic acid based on body weight via the tail vein: 10 μg/g, 25 μg/g, or 50 μg/g. The control group was injected with an equal volume of DMSO. Samples were collected on the seventh day after injection. After anesthetizing the fish using MS-222, skin and eye tissues were collected, and they were subsequently snap-frozen in liquid nitrogen and stored at −80 °C until further processing. The expression levels of skin-color-related genes were detected by qPCR, and the determination of pigments, MSH content, and tyrosinase activity were assessed using ELISA kits (Zhenke Industrial International, Zhongshan, China) following the manufacturer’s instructions.

### 4.9. Statistical Analysis

Statistical analysis of mRNA expression differences was conducted using IBM SPSS Statistics 22.0 (SPSS Inc., Chicago, IL, USA; an IBM Company, Amonk, NY, USA) software with one-way ANOVA. Data are expressed as mean ± standard error. Duncan’s multiple range tests were employed to discern differences between experimental groups. Assumptions of normality and equal variance were met for all parameters, and the statistical analyses confirmed the significant differences (*p* < 0.05) between the experimental groups.

## 5. Conclusions

In summary, our study uncovered the crucial role that the *sws*2 gene plays in regulating skin color differentiation in *P. leopardus*. We successfully isolated the *sws*2 gene from *P. leopardus* and detected its mRNA expression in all tissues. Its localization in the eye and skin tissues suggests that it plays an important role in regulating skin color changes. *Sws*2 gene knockdown led to significant decreases in the expression of black-color-related genes, melanin contents, and tyrosinase activity in the skin, and increases in *scarb*1 gene expression and carotenoid and lutein contents, while retinoic acid injection produced the opposite results. These results suggest that the *sws*2 gene is involved in *P. leopardus* skin color regulation by affecting vitamin synthesis and melanin-related gene expression levels. Our findings offer novel insights into the molecular mechanisms underlying skin color differentiation in fish, laying a foundation for comprehending how *sws*2 regulates melanocyte formation in fish skin at the molecular level.

## Figures and Tables

**Table 1:** Primer sequences.

Primer	Sequence (5′-3′)	Application	Gene Name	Accession Number
sws2 RACE F1 (first)	TCTTTGGGTCGTCAACAACCG	sws2 cloning	-	-
sws2 RACE R1 (first)	GAGGTGCCCGCAGTGAATAA		-	-
sws2 RACE F2 (nested)	GCGGGCAGCCTTTTGACCT		-	-
sws2 RACE R2 (nested)	AGGAAGGGGCTGAAAGCTGT		-	-
mc1r F	TGCCAGAACCACCAGGAT	Quantitative RT-PCR	melanocortin 1 receptor	XM042485859.1
mc1r R	ACGGAAACGACCAACAGG			
sws2 F	GTTGTCGGTGGTGTACCAGT		-	-
sws2 R	GGGTGTTCGCACTAATTGCC		-	-
tyr F	GTCTTCAACATCCTCAGCGGT		tyrosinase	XM042486951.1
tyr R	GGTCGCATAGACAGTGCTTCC			
tyrp1 F	CGTAAGAGTAGCCAAGATTTTCA		tyrosinase related protein 1	Transcriptome data
tyrp1 R	GTTGAGGAGACACAGTCCAGAT			
pomc F	GTCGAGATCTGACGGAGGAG		proopiomelanocortin	Transcriptome data
pomc R	AGTCAGTGCTGGGAACATCC			
Scarb1 F	ACCAGTCCGCTGTCATAACC		scavenger receptor class B, member 1	XM042509253.1
Scarb1 R	CACCGTGTCCTACAGGGAGT			
β-actin F	TCTGGGCAACGGAACCTCT		beta-actin mRNA	OK483033.1
β-actin R	CACCACAGCCGAGAGGGA			

## Data Availability

Data is contained within the article.
